# Evaluating the mental health impacts of an anti-racism intervention for children and young people

**DOI:** 10.1186/1475-9276-11-S1-A5

**Published:** 2012-01-23

**Authors:** Margaret Kelaher, Yin Paradies, Deborah Joy Warr, Angeline Ferdinand, Teneha Greco, Kaitlin Lauirdsen

**Affiliations:** 1 Centre for Health Policy, Programs and Economics, Melbourne School of Population Health, University of Melbourne, Vic, 3010, Australia; 2 McCaughey Centre, VicHealth Centre for the Promotion of Mental Health and Community Wellbeing Melbourne School of Population Health, University of Melbourne, Vic, 3010, Australia

## Background

The Building Bridges initiative was designed to promote positive cross-cultural interactions among young people and improve the mental health of migrant and refugee populations by addressing race-based discrimination. The goal of the evaluation was to build the evidence and knowledge base for promoting mental health and wellbeing by reducing discrimination through promoting cooperative intercultural contact. The design of the evaluation was multi-level with data collected at the individual, organisational and community levels to evaluate change associated with program participation and examine barriers and enablers to implementation.

## Methods

The evaluation used a before and after design. It examined characteristics of participants, the environment for intercultural contact (equal status between ethnic groups, shared goals, co-operation and institutional support for intercultural relationships), attitudes to people from other cultures and Basic Psychological Needs [1]. Both quantitative (pre: n=436 and post: n=686) and qualitative data (sessions=14 and n=170) were collected.

## Results

Building Bridges was effective in reaching children and young people who were affected by discrimination and for whom improving intercultural relations was a priority. Building Bridges projects all scored highly on their ability to provide an environment for effective intercultural contact. The results suggest that performance against the Equal treatment and Shared goals/Co-operation is important for how participants feel about themselves and others. Institutional Support for intercultural contact may be critical for translating these gains into meaningful changes in intercultural relationships.

The analysis of the impact of the intervention showed that attitudes to intercultural relationships remained the same or improved marginally for people with nationalities other than Australian over the course of the intervention. However, attitudes to intercultural relationships declined for people with Australian ethnicity bringing them in closer alignment with the attitudes of participants with nationalities other than Australian. There was also evidence of positive effects on mental wellbeing with particular improvements for people with ethnicities other than Australian. Overall, the results suggest of a shift in power relations between the populations involved which had positive health effects.

## Conclusions

Overall, Building Bridges is a pilot program that has benefited the individuals, organisations and community involved and has yielded important learnings about the implementation of pro-diversity programs. The evaluation findings challenged assumptions about the impact of living in a multicultural society on intercultural relations and make a significant contribution to our understanding of pro-diversity interventions.
